# Thorax radiotherapy using ^18^F-positron emission tomography/computed tomography-guided precision radiotherapy is a prognostic factor for survival in patients with extracranial oligometastatic non-small cell lung cancer:A two-center propensity score-matched analysis

**DOI:** 10.3389/fonc.2022.991378

**Published:** 2022-10-18

**Authors:** Cheng-Sen Liu, Ying-Qiu Song, Run-Ze Wang, Zheng Wang, Rong He, Ke Xu, Chen-Yu Wang, Yu Wu, Ye Wang, Xiao-fang Zhang, Guang Li, Tian-Lu Wang

**Affiliations:** ^1^ Department of Radiotherapy, Cancer Hospital of China Medical University, Liaoning Cancer Hospital and Institute, Shenyang, Liaoning, China; ^2^ Department of Thoracic Surgery, Liaoning Cancer Hospital and Institute, Shenyang, Liaoning, China; ^3^ Department of Information Management, Liaoning Cancer Hospital and Institute, Shenyang, Liaoning, China; ^4^ Department of Radiotherapy, The First Hospital of China Medical University, Shenyang, Liaoning, China

**Keywords:** positron emission tomography/computed tomography, thorax radiotherapy, prognosis, extracranial oligometastatic, non-small cell lung cancer

## Abstract

**Background:**

This retrospective study compared positron emission tomography (PET)/computed tomography (CT) and CT in the treatment of extracranial oligometastatic non-small-cell lung cancer (NSCLC) and explored the impact of thorax radiotherapy (TRT) on patient survival.

**Methods:**

We reviewed the medical records of Chinese patients with stage IV extracranial oligometastatic NSCLC who underwent PET/CT or CT at two centers. Propensity score matching (PSM) was used to control differences in patient characteristics between the maintenance chemotherapy alone and TRT plus maintenance chemotherapy groups.

**Results:**

We analyzed 192 eligible patients. The median survival time was better in patients who received PET/CT than in those who only received CT (n = 192, 16 months vs. 6 months, *p*<0.001). Subgroup analysis showed the median survival time was significantly longer in the TRT plus maintenance group than in the chemotherapy alone group in patients who underwent PET/CT examinations (n = 94, 25 months vs. 11 months, *p*<0.001). However, there was no statistical difference in survival between both groups in patients who underwent CT examinations (n = 98, 8 months vs. 5 months, *p* = 0.180). A multifactorial analysis revealed a more favorable prognosis in patients who underwent PET/CT evaluation (HR: 0.343, 95% CI: 0.250-0.471, *p <*0.001) and TRT (HR: 0.624, 95% CI: 0.464-0.840, *p* = 0.002), than in those who did not. PSM was consistent with these results.

**Conclusions:**

PET/CT-guided TRT is associated with improved clinical outcomes in patients with stage IV extracranial oligometastatic NSCLC.

## Introduction

Oligometastasis is a distant metastasis with a limited number and distribution of tumors. Patients with oligometastasis usually have a maximum of 3-5 metastatic sites, excluding the primary site, and no more than 3 different organs that can be treated locally using surgery, radiation therapy, and other treatments to improve survival (1). Currently, oligometastasis is considered the transitional stage of advanced tumors inducing limited to extensive metastases, with the former metastases having significantly different biology and treatment options than those of the latter metastases (2). This is a shift from the conventional oncological thinking, which considers metastasis as an end-stage disease with limited treatment options. In patients with non-small-cell lung cancer (NSCLC) receiving systemic therapy such as chemotherapy, the most common progression site is the original disease site (3–6). Our previous studies have shown that aggressive local consolidation therapy may destroy lesions, slow disease progression, and even improve patient survival (7). The results of a recent meta-analysis showed that combination local therapy in patients with metastatic NSCLC improved patient progression-free survival (PFS) and overall survival (OS) and did not increase grade ≥3 adverse events. These results are consistent with the encouraging findings of most current randomized studies in the context of the lack of large sample phase 3 randomized studies, and support the exploration of local therapy in the treatment of metastatic NSCLC with the aim of achieving a cure (8).

Radiation therapy is one of the main local advanced NSCLC treatments. The clinical outline of the gross tumor volume (GTV) is based on morphological information, such as tissue density and anatomical structure, as provided by computed tomography (CT), but not on corresponding metabolic information. Consequently, potential occult metastases are often undetected, and GTV depiction based on planned CT images can be difficult when there is limited contrast between the tumor and the surrounding tissue with similar density, such as in cases of mediastinal invasion or pulmonary atelectasis (9). ^18^F-fluorodeoxyglucose positron emission tomography-CT (^18^FDG-PET/CT) is of great value for early lung cancer detection, differential diagnoses of isolated nodules in lungs and mediastinum lymph nodes, clinical staging and detection of distant metastases, treatment efficacy determination, recurrence and metastasis follow-ups, and prognosis (10, 11). ^18^FDG, the most widely used PET imaging agent in clinical practice, enters the cell, following which ^18^FDG-PET imaging distinguishes between benign and malignant lesions based on glucose metabolism, which is significantly correlated with tumor pathological response (12). Most lung cancers are highly metabolic in nature, and abnormal cancer cell proliferation requires glucose overutilization. Accordingly, ^18^FDG-PET/CT has the advantages of both functional and anatomical structural imaging. ^18^FDG-PET/CT is better than traditional imaging methods and is increasingly preferred by clinicians for the diagnosis, treatment, and treatment efficacy evaluation of lung cancer and other tumors (13–15). Combining PET imaging with radiation therapy facilitates molecular image-guided dose mapping strategies that can create non-uniform dose distributions within the target area (16). To this end, PET can be used to visualize and quantify specific biological processes, including glucose metabolism, cell proliferation, and hypoxia, which are closely related to the radiotherapy process (17).

We demonstrated that local consolidation therapy (LCT) is superior to maintenance therapy in patients with oligometastatic NSCLC receiving PET/CT guidance (7). However, we did not directly compare NSCLC patients who underwent PET/CT to those who underwent conventional CT, and there is no direct evidence to confirm a significant difference in the prognostic benefit between these two screening methods in patients with oligometastatic NSCLC. Undertreatment may significantly contribute to treatment failure or tumor recurrence, but overtreatment can cause serious side effects and physical damage. Minimizing unnecessary treatment while ensuring efficacy is important for the survival and quality of life of patients with advanced NSCLC. To further improve the diagnosis and treatment of oligometastatic NSCLC, we retrospectively studied the medical records of patients with extracranial oligometastatic NSCLC who underwent PET/CT and conventional CT at two centers to investigate the differences between both examination methods and the impact of radiation therapy on the primary lung cancer site and on the survival of patients with extracranial oligometastatic NSCLC.

## Materials and methods

### Study population

We retrospectively analyzed Chinese patients with stage IV extracranial oligometastatic NSCLC who had undergone PET/CT or CT scans at the First Hospital of China Medical University and Liaoning Cancer Hospital from February 2013 to November 2019. From patients’ medical records, we assessed age, sex, smoking history, patient status, tumor size, lymphatic metastases, histological type, primary tumor site, treatment choice, and date of death. Inclusion criteria were patients with extracranial oligometastatic NSCLC with complete medical records who received thorax radiotherapy (TRT) with systemic therapy or only systemic therapy. PET/CT or CT was conducted on selected patients within one month before treatment. Patients with major organ dysfunction, more than one primary tumor, unknown metastatic status, multiple metastases (number >5), malignant pleural effusion, or who underwent targeted therapy or immunotherapy were excluded.

Propensity score matching ((PSM), performed using KPS、Smoke、Position、N classification, and Weight loss) was used to create a maintenance chemotherapy alone group, and a thorax radiotherapy (TRT) plus maintenance chemotherapy group, to reduce the effects of selection bias and confounding variables. The PSM function of the IBM SPSS software (IBM Co., Armonk, NY, USA) was used to estimate the propensity score, and 1:1 nearest neighbor matching was used with a caliper width of 0.02 for PSM. A Chi-square test was used to check the covariate balance of each subgroup before and after PSM.

### Statistical analysis

The number and clinical characteristics of all eligible patients were documented, and the Chi-square test was used to compare the categorical variables. The time from each patient’s first extracranial oligometastatic diagnosis to the last follow-up or death was recorded as overall survival time (OST). The survival curves of different variables were determined using the Kaplan–Meier method and then checked by a log-rank test. The results are reported as hazard ratios (HRs) and 95% confidence intervals (CIs). Forest plots were used to describe the subgroup analysis of all the data. From the univariate analysis results, significant variables (*p*<0.1) were selected and placed in a Cox regression model and then a multivariable analysis, to ensure that significant variables (*p*<0.05) associated with the prognosis of patients with extracranial oligometastatic NSCLC were identified. IBM SPSS was used for the statistical analysis, and GraphPad Prism8 was used to draw the survival curve and forest plots.

## Results

### Patient characteristics

**Figure 1:**
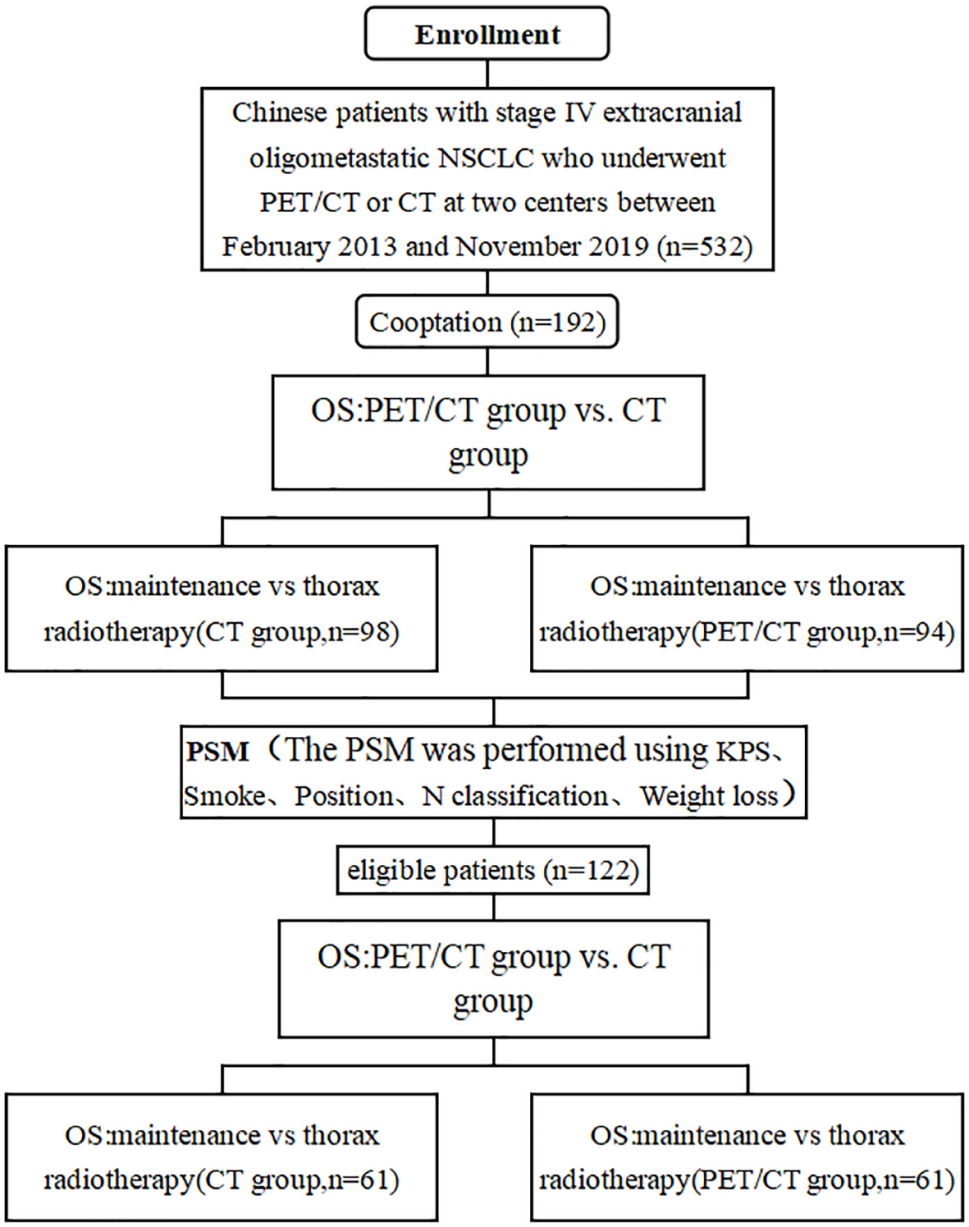
Flowchart depicting selection of the study population.

For the analysis, 192 of a total of 532 patients with advanced NSCLC at two medical centers were selected; 113 patients were male, and 79 were female, with a median age of 60 years. We used PSM to select 122 eligible patients of the 192 patients with extracranial oligometastatic NSCLC and divided them into a CT group and a PET/CT group (**Figure 1**). Among all patients, the one-year and two-year overall survival rates were 41.7% and 15.1%, respectively, and the median survival time was 10.5 months. Patient demographic and clinical characteristics are presented in **Table 1**.

**Table 1:** Baseline characteristics of patients.

Characteristics	BEFORE PSM			AFTER PSM		
	CT (N=98)	PET/CT (N=94)	*P* value	CT (N=61)	PET/CT (N=61)	*P* value
Age (years)						
<60	53	40	0.110	34	26	0.147
≥60	45	54		27	35	
Sex						
Female	35	44	0.118	28	27	0.856
Male	63	50		33	34	
KPS						
<90	43	57	0.002	31	31	1
≥90	55	37		30	30	
Smoke						
No	47	66	0.002	36	38	0.711
Yes	51	28		25	23	
Pathological pattern						
SQC	43	29	0.062	28	21	0.196
Adenocarcinoma and others	55	65		33	40	
Position						
Central	51	67	0.006	40	39	0.850
Peripheral	47	27		21	22	
T classification						
T1-2	39	43	0.405	23	28	0.359
T3-4	59	51		38	33	
N classification						
N0-1	18	32	0.013	14	13	0.827
N2-3	80	62		47	48	
Therapy						
No TRT	46	50	0.386	35	32	0.585
TRT	52	44		26	29	
Weight loss						
≤5%	74	61	0.026	40	40	1
>5%	24	33		21	21	
Number of metastasis						
1	32	23	0.540	16	15	0.770
2	24	23		17	17	
3	14	19		9	10	
4	18	15		13	9	
5	10	14		6	10	
No. of metastatic organs						
1	56	46	0.384	33	32	0.389
2	32	33		22	18	
3	10	15		6	11	
Lung metastasis	48	49	0.663	29	32	0.587
Bone metastasis	44	46	0.575	30	28	0.717
Liver metastasis	11	11	0.917	7	7	1
Adrenal metastasis	5	5	1	3	1	0.619
Other metastasis	42	46	0.398	26	33	0.205
Mixed metastasis	41	48	0.200	27	29	0.716

N, number of cases/controls; TRT, thorax radiotherapy; PSM, propensity score matching; The PSM was performed using KPS, Smoke, Position, N classification, Weight loss; which were subdivided according to the median values; AD, adenocarcinoma.

### Univariate analysis

Considering tumor heterogeneity, we did not examine the relationship between different chemotherapy regimens and survival time. Important variables included in the univariate analysis before and after PSM, including T and N classifications, image (PET/CT vs. CT) and therapy, (TRT vs. NO TRT) are presented in **Tables 2** and **3** (all *p*<0.1). The median survival time was better in patients who received PET/CT than in those who only received CT (before PSM: 16 months vs 6 months; and after PSM: 19 months vs 6 months, both, *p*<0.001). The median survival time was significantly better in the TRT plus maintenance group than in the chemotherapy alone group (before PSM: 13 months vs 8 months, *p*<0.001; and after PSM: 14 months vs 8 months, *p*<0.001).

**Table 2:** Univariate analysis between prognostic factors and overall survival (Before PSM).

Characteristics	N	Median survival time (months)	1-year os (%)	2-years os (%)	X^2^	*P* value
Age(years)						
<60	93	11	39.8	15.1	0.004	0.950
≥60	99	10	43.4	15.1		
Sex						
Female	79	11	44.3	20.3	2.382	0.123
Male	113	9	39.8	11.5		
KPS						
<90	100	10	39.0	44.6	0.033	0.855
≥90	92	11	17.0	18.5		
Smoke						
No	113	10	39.8	15.0	0.026	0.872
Yes	79	11	44.3	15.2		
Pathological pattern						
SQC	72	10	38.9	9.7	1.308	0.253
Adenocarcinoma and others	120	10	43.3	18.3		
Position						
Central	118	12	46.6	18.6	2.536	0.111
Peripheral	74	8	33.8	9.5		
T classification						
T1-2	82	12	48.8	19.5	3.819	0.051
T3-4	110	9	36.4	11.8		
N classification						
N0-1	50	15	56.0	24.0	7.560	0.006
N2-3	142	10	36.6	12.0		
Image						
CT	98	6	25.3	2.0	58.629	<0.001
PET/CT	94	16	63.8	28.7		
Weight loss						
≤5%	135	11	43.7	12.6	0.204	0.651
>5%	57	10	36.8	21.1		
Thorax radiotherapy						
No	96	8	32.2	6.3	13.118	<0.001
Yes	96	13	51.0	24.0		
Number of metastasis						
1	55	10	41.8	16.4	1.635	0.802
2	47	9	42.6	12.8		
3	33	12	45.5	12.1		
4	33	9	36.4	21.2		
5	24	11	41.7	12.5		
No. of metastatic organs						
1	102	10	42.2	14.7	0.289	0.865
2	65	11	41.5	16.9		
3	25	11	40.0	12.0		
Lung metastasis	97	11	45.4	17.5	1.499	0.221
Bone metastasis	90	11	38.9	16.7	0.021	0.884
Liver metastasis	22	9	36.4	13.6	0.340	0.560
Adrenal metastasis	10	5	40.0	0	4.143	0.042
Other metastasis	88	11	40.9	12.5	0.458	0.498
Mixed metastasis	89	11	41.6	15.7	0.026	0.871

N, number of cases/controls; PSM, propensity score matching.

**Table 3:** Univariate analysis between prognostic factors and overall survival (After PSM).

Characteristics	N	mediansurvival time (months)	1-year os(%)	2-years os(%)	X2	P value
Age(years)						
<60	60	11	41.7	15.0	0.306	0.580
≥60	62	9	40.3	19.4		
Sex						
Female	55	11	40.0	20.0	0.040	0.841
Male	67	10	41.8	14.9		
KPS						
<90	62	9	35.5	14.5	0.030	0.863
≥90	60	11	46.7	20.0		
Smoke						
No	74	10	39.2	16.2	0.066	0.798
Yes	48	11	43.8	18.8		
Pathological pattern						
SQC	49	11	42.9	14.3	0.059	0.809
Adenocarcinoma and others	73	10	39.7	19.2		
Position						
Central	79	11	43.0	17.7	0.120	0.729
Peripheral	43	9	37.2	16.3		
T classification						
T1-2	51	12	47.1	19.6	0.955	0.329
T3-4	71	9	36.6	15.5		
N classification						
N0-1	27	14	55.6	25.9	3.086	0.079
N2-3	95	10	36.8	14.7		
Image						
CT	61	6	14.8	3.3	41.656	<0.001
PET/CT	61	19	67.2	31.1		
Weight loss						
≤5%	80	11	47.5	16.2	0.964	0.326
>5%	42	8	28.6	19.0		
Thorax radiotherapy						
No	67	8	29.8	6.0	15.448	<0.001
Yes	55	14	54.5	30.9		
Number of metastasis						
1	31	11	41.9	16.1	0.542	0.969
2	34	9	41.2	17.6		
3	19	11	42.1	21.1		
4	22	10	36.4	18.2		
5	16	11	43.8	12.5		
No. of metastatic organs						
1	65	10	41.5	16.9	0.488	0.783
2	40	11	40.0	20.0		
3	17	11	41.2	11.8		
Lung metastasis	61	11	45.9	18.0	0.415	0.519
Bone metastasis	58	11	41.4	19.0	0.279	0.597
Liver metastasis	14	8	35.7	21.4	0.128	0.720
Adrenal metastasis	4	5	0	0	9.191	0.002
Other metastasis	59	11	39.0	13.6	0.064	0.800
Mixed metastasis	56	11	41.1	17.9	0.313	0.576

N, number of cases/controls; PSM, propensity score matching.

### Survival analysis and subgroup analysis

**Figure 2:**
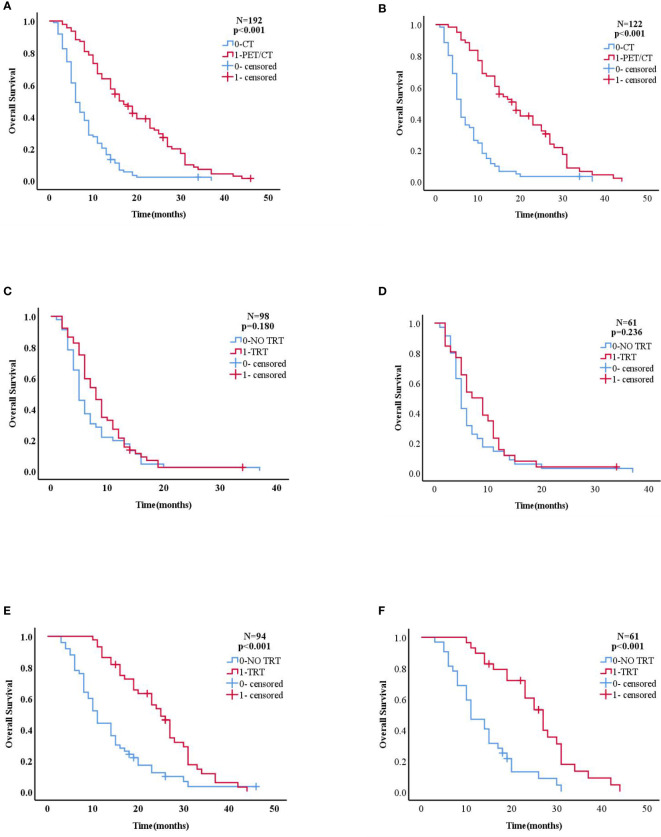
**(A, B)** Kaplan–Meier curve of overall survival for patients who did and did not receive PET/CT in the overall population (before PSM n=192,after PSM n=122). **(C, D)** Kaplan–Meier curve of overall survival for patients who did and did not receive TRT in the CT group (before PSM n=98, after PSM n=61). **(E, F)** Kaplan–Meier curve of overall survival for patients who did and did not receive TRT in the PET/CT group(before PSM n=94,after PSM n=61).

**Figure 3:**
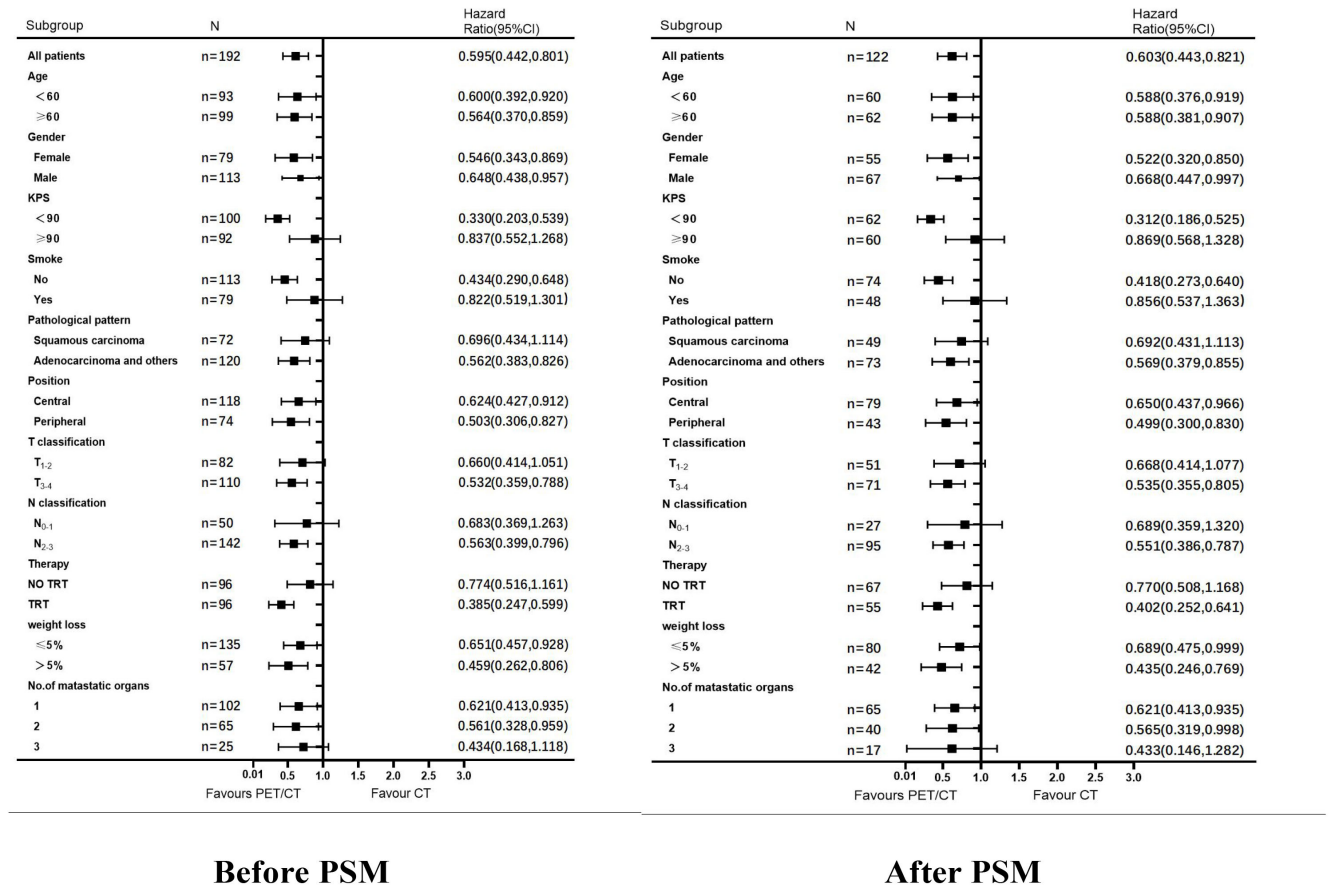
Analysis of OS among patients in the PET/CT group VS. CT group. Subgroup analysis of OS among patients in the PET/CT group TRT VS. No TRT.

**Figures 2** and **3** show that patients who underwent PET/CT were significantly associated with improved survival times. A subgroup analysis for those who underwent PET/CT examination, found a significant difference between the aforementioned groups: before PSM, 25 months vs 11 months; and after PSM, 27 months vs 11 months, (both, *p*<0.001). However, no statistical difference in survival was found between the chemotherapy alone and the TRT plus maintenance groups for those who underwent CT examination: before PSM, 8 months vs 5 months, *p* = 0.180; and after PSM, 7 months vs 5 months, (*p* = 0.236).

### Multifactorial analysis

Factors with significant differences in univariate analysis were subjected to multifactorial analysis. In the multifactorial analysis (**Table 4**), patients who underwent PET/CT (before PSM: HR: 0.343, 95% CI: 0.250–0.471 and after PSM, HR: 0.323, 95% CI: 0.217–0.480, (both *p <*0.001) and TRT before PS,: HR: 0.624, 95% CI: 0.464–0.840, (*p* = 0.002) and after PSM, HR: 0.569, 95% CI: 0.385–0.841, (*p* = 0.005), had a more favorable prognosis than those who underwent CT and chemotherapy alone.

**Table 4:** Multivariate analysis between prognostic factors and overall survival.

Factor	Before PSM			After PSM		
	HR	95% CI	*P* value	HR	95% CI	*P* value HR
T classification (T1-2 vs.T3-4)	1.138	0.830-1.561	0.422	0.915	0.606-1.380	0.670
N classification (N0-1vs. N2-3)	1.268	0.872-1.843	0.214	1.493	0.899-2.481	0.121
Image (CT vs. PET/CT)	0.343	0.250-0.471	<0.001	0.323	0.217-0.480	<0.001
Therapy (No TRT vs. TRT)	0.624	0.464-0.840	0.002	0.569	0.385-0.841	0.005

## Discussion

Traditional NSCLC staging is based primarily on history, fiberoptic bronchoscopy, CT, magnetic resonance imaging, and other routine imaging examinations. If the lung cancer stage is inaccurate, an inappropriate treatment regimen will inevitably have an adverse impact on patient survival. PET/CT is an advanced diagnostic medical imaging technique that shows local metabolic function at a refined anatomical level. It has a superior sensitivity and specificity to other examination methods, and allows a more effective detection of asymptomatic metastatic foci and metastatic lymph nodes (18). Staging is more accurate when both metabolic function and anatomical information are assessed (19). CT imaging may be less applicable in classifying patients as oligometastases due to its relatively less sensitive imaging. Because occult lesions are not detected during CT imaging, the number of metastases recorded is less than the true picture (20, 21). This can lead to the misclassification of patients with extensive metastases as those who develop oligometastases, and the limited use of local therapy for this group of patients, which may be one of the reasons why the prognosis of patients who received PET/CT-guided thorax radiotherapy in our study was better than that of patients who underwent CT examination. PET/CT can also improve the accuracy of depicting the target area for radiotherapy, avoiding missed target areas or increased exposure. Using PET/CT during an NSCLC radiotherapy program improves target volume delineation reliability and allows higher radiation doses without increasing the risks of side effects (22). Accordingly, the volume of the scheduled irradiation target can be more accurately limited to morphologically and functionally defined tumor areas. Fewer normal tissues can be irradiated and higher total tumor doses can be used to seek optimal therapeutic effects and develop more rational treatments. Studies report that occult extra-thoracic metastases can be found in up to 37% of patients with advanced NSCLC, altering 14%-26% of NSCLC treatment decision (23). PET/CT has become the standard imaging tool for characterizing lung nodules (24), initial staging (25, 26), treatment planning, treatment response assessment (27), recurrence staging (28, 29), and lung cancer monitoring. The widespread clinical use of FDG-PET/CT in patients with lung cancer has improved staging and restaging accuracies, allowing for better treatment planning and response to treatment assessments. To further explore the role of PET/CT in the treatment of extracranial oligometastases, we compared the prognosis of patients who underwent PET/CT and CT. We found that PET/CT-localized TRT improved survival, while CT imaging-guided radiotherapy did not, indicating a significant advantage in the use of PET/CT in the extracranial oligometastatic NSCLC therapeutic schedule. To our knowledge, this is the first study to report the significance of PET/CT compared with CT in guiding TRT for extracranial oligometastatic NSCLC, and the findings need to be validated by further expansion of the sample size and prospective studies.

Local consolidation therapy (LCT) is a common treatment for oligometastatic NSCLC. In 2016, Gomez et al. (30) were the first to report the results of a phase II randomized trial that compared standard maintenance therapy (n = 24) to LCT (n = 25). The median patient follow-up was 12.39 months, and the PFS was significantly better in the maintenance treatment group than in the local consolidation group (11.9 months vs. 3.9 months, *p* = 0.0054). Adverse events were similar between both groups, with no treatment-induced grade 4 adverse events or deaths. Gomez et al. (31) reported the results of their latest long-term clinical study in 2019 and confirmed that PFS (median, 14.2 months [95% CI, 7.4 to 23.1 months] with LCT, vs. 4.4 months [95% CI, 2.2 to 8.3 months] with maintenance therapy or observation; *p* = 0.022) and overall survival (median, 41.2 months [95% CI, 18.9 months to not reached] with LCT, vs. 17.0 months [95% CI, 10.1 to 39.8 months] with maintenance therapy or observation, (*p* = 0.017) was significantly improved by the early inclusion of LCT. They also reported that both initial LCT before progression and delayed LCT after progression contributed to improved overall survival. In 2018, Iyengar et al. reported results from a phase II randomized trial comparing standard maintenance therapy with and without stereotactic ablative radiotherapy (SABR) in a patient population nearly identical to that of the trial population reported by Gomez et al. They showed that PFS was significantly better in the SABR plus maintenance chemotherapy group, than in the maintenance chemotherapy alone group (9.7 months vs. 3.5 months, *p* = 0.01) (6). Gomez et al. and Iyengar et al. both conducted phase II trials in patients with oligometastatic NSCLC to study the prognostic impact of aggressive local therapy. Both trials reported a significant increase in PFS by increasing aggressive local therapy. Moreover, our previous study showed that PET/CT-guided LCT was significantly effective in patients with oligometastatic advanced lung cancer [7]. Overall survival rates were much higher in the LCT group than in the chemotherapy alone group (13 months vs. 7 months, (*p* = 0.002). The incidence of side effects was similar between the LCT and chemotherapy alone groups, and there were no treatment-related adverse outcomes or deaths. To explore more effective and personalized treatment options for oligometastatic lung cancer, we focused on evaluating the efficacy of extracranial oligometastases treated with PET/CT vs. CT guided TRT and revealed that similar efficacy was achieved with PET/CT while CT was less effective, meaning that it is possible to achieve satisfactory outcomes with treatments and that PET/CT guides a more precise thorax radiotherapy regimen to patients with extracranial oligometastatic NSCLC than that of CT. There are several possible mechanisms that could explain the survival benefits of TRT. First, after systemic treatment, hard-to-treat malignant cells, which are unlikely to be eliminated by subsequent maintenance therapy and can serve as a source of metastatic spread, are left behind. However, in such cases, TRT may reduce the number of such cells. Second, TRT may enhance the effects of systemic therapy by possibly making residual lesions more sensitive to subsequent maintenance therapy. A third possibility is that radiotherapy kills tumor cells by modulating the immune system. Radiotherapists may be subjective in the process of outlining tumor target areas based on CT images, and the influence of experience of PET/CT on the accuracy of tumor target area outlining can further affect the efficacy of radiotherapy. In particular, patients with advanced lung cancer often have complications such as pulmonary atelectasis, which is difficult to identify with the tumor lesions. Therefore, we believe that CT plays a limited role in the process of target area outlining, and it is difficult to improve the prognosis of patients with lung cancer. A well-defined lesion is shown in PET/CT, which can exclude the influence of subjective factors on the accuracy of target area outlining and thus improve the efficacy of radiotherapy.

Immunotherapy was not included in this study because the patient information we analyzed was first obtained in 2013. As research progresses, it is increasingly recognized that there are complex interactions between radiotherapy and the immune system. In addition to producing a local therapeutic effect at the irradiated tumor site, radiotherapy can also cause spontaneous tumor regression in non-irradiated lesions; this is known as the abscopal effect (32). Although the abscopal effect was studied for decades, the exact mechanism of this phenomenon is still unclear, and Demaria et al. (33) first linked the distal effect of radiotherapy to an immune-mediated mechanism. Preclinical studies suggest that radiotherapy is equivalent to an “agonist” in immunotherapy, making tumor cells more susceptible to T-cell-mediated immune attacks by modulating the immune system. Radiotherapy can enhance anti-tumor immune effects by inducing the release of more neoantigens from damaged tumor cells, enhancing the expression of major histocompatibility complex class I molecules, and upregulating chemokines, cell adhesion molecules, and other immunomodulatory cell surface molecules, thereby inducing immunogenic cell death (34). In terms of increasing attention to immunotherapy, the possibility of combining radiotherapy with immunotherapy is worth exploring, and this combination to produce synergistic antitumor activity shows great application prospects and development potential in the future.

## Conclusions

PET/CT-guided TRT is associated with improved clinical outcomes in patients with stage IV extracranial oligometastatic NSCLC when compared to that of CT. Advances in radiotherapy technology have improved radiotherapy precision and effectiveness while ensuring treatment safety. Optimization and innovation of radiotherapy technology in positioning, target area outlining, planning, and beam projection will continue to be a strong driving force for applying and developing radiotherapy in patients with lung cancer. Future basic research and large-sample randomized clinical trials are required to optimize further combinations of radiotherapy and systemic therapy, specifically in terms of targeted therapy and immunotherapy for patients with NSCLC.

## Data availability statement

The original contributions presented in the study are included in the article/supplementary material. Further inquiries can be directed to the corresponding author.

## Ethics statement

Ethical review and approval was not required for the study on human participants in accordance with the local legislation and institutional requirements. Written informed consent from the (patients/participants OR patients/participants legal guardian/next of kin) was not required to participate in this study in accordance with the national legislation and the institutional requirements.

## Author contributions

CSL, YQS and RZW; formal analysis: CSL and YQS; data curation: RH and KX; software management: CYW; project administration: ZW; validation: GL; visualization: YWu, YWa and XfZ; draft review & editing: all authors; funding acquisition: TLW, YQS, additional resources: TLW.

## Funding

This study has received funding by the Cancer Research Program of National Cancer Center [NCC2017A08], the Liaoning Province Natural Science Foundation [2020-ZLLH-47], the Liaoning Province Natural Science Foundation [2020-MS-065], the Liaoning Province Natural Science Foundation [2017225054], the Fundamental Research Funds for the Central Universities [LD202127, LD202221], Joint fund of Science & Technology Department of Liaoning Province and State Key Laboratory of Robotics, China [2019-KF-01-01], the Tumor Mass spectrometry Center project [ZP202013, ZP202008], Key Laboratory of Tumor Radiosensitization and Normal Tissue Radioprotection Project of Liaoning Province [2018225102], Liaoning Provincial Medical and Industrial Cross-Joint Fund [2021-YGJC-02].

## Acknowledgments

We would like to thank all patients and their families for their active cooperation and all hospital staff for their assistance in the study.

## Conflict of interest

The authors declare that the research was conducted in the absence of any commercial or financial relationships that could be construed as a potential conflict of interest.

## Publisher’s note

All claims expressed in this article are solely those of the authors and do not necessarily represent those of their affiliated organizations, or those of the publisher, the editors and the reviewers. Any product that may be evaluated in this article, or claim that may be made by its manufacturer, is not guaranteed or endorsed by the publisher.
